# Design of an Electronic Interface for Single-Photon Avalanche Diodes

**DOI:** 10.3390/s24175568

**Published:** 2024-08-28

**Authors:** Salvatore A. Pullano, Giuseppe Oliva, Twisha Titirsha, Md Maruf Hossain Shuvo, Syed Kamrul Islam, Filippo Laganà, Antonio La Gatta, Antonino S. Fiorillo

**Affiliations:** 1 Department of Health Sciences, “Magna Graecia” University, 88100 Catanzaro, Italy; giuseppe.oliva@unicz.it (G.O.); filippo.lagana@unicz.it (F.L.); tsem.spa@gmail.com (A.L.G.); nino@unicz.it (A.S.F.); 2 Department of Electrical Engineering and Computer Science, University of Missouri, Columbia, MO 65211, USA; ttkff@missouri.edu (T.T.); islams@missouri.edu (S.K.I.); 3 Department of Electrical and Computer Engineering, University of Texas at El Paso, El Paso, TX 79968, USA; mhshuvo@utep.edu

**Keywords:** avalanche transistor, quenching circuits, single-photon avalanche diode (SPAD), single-photon timing resolution

## Abstract

Single-photon avalanche diodes (SPADs) belong to a family of avalanche photodiodes (APDs) with single-photon detection capability that operate above the breakdown voltage (i.e., Geiger mode). Design and technology constraints, such as dark current, photon detection probability, and power dissipation, impose inherent device limitations on avalanche photodiodes. Moreover, after the detection of a photon, SPADs require dead time for avalanche quenching and recharge before they can detect another photon. The reduction in dead time results in higher efficiency for photon detection in high-frequency applications. In this work, an electronic interface, based on the pole-zero compensation technique for reducing dead time, was investigated. A nanosecond pulse generator was designed and fabricated to generate pulses of comparable voltage to an avalanche transistor. The quenching time constant (τ_q_) is not affected by the compensation capacitance variation, while an increase of about 30% in the τ_q_ is related to the properties of the specific op-amp used in the design. Conversely, the recovery time was observed to be strongly influenced by the compensation capacitance. Reductions in the recovery time, from 927.3 ns down to 57.6 ns and 9.8 ns, were observed when varying the compensation capacitance in the range of 5–0.1 pF. The experimental results from an SPAD combined with an electronic interface based on an avalanche transistor are in strong accordance, providing similar output pulses to those of an illuminated SPAD.

## 1. Introduction

Photon detection using single-photon avalanche diodes (SPADs) has been investigated as a highly sensitivity alternative to photomultiplier tubes, silicon photomultipliers, etc., in manifold applications. The most recent application of SPADs in medicine has focused on DNA analysis, protein dynamics measurements using light scattering, two-photon fluorescence microscopy, automated DNA sequencing machines, advanced positron emission tomography, particle and drop sizing, optical biopsy, implantable and endovascular fluorescence probes, etc. [1,2,3,4]. Furthermore, the development of configurable SPAD models with intelligent control can provide an overall detector response and enable performance analysis [5,6]. Due to strict requirements in terms of spectral sensitivity and temporal resolution, down to a few tens of picoseconds (ps), solid-state photodetectors have been amply investigated [7,8]. The investigated photodiodes operate above the breakdown voltage (i.e., Geiger mode) and can be realized with reverse-biased p-n junctions, in which an incident photon can trigger avalanche multiplication of the generated electron–hole pairs. The current drawing is limited by the external circuit and can reach the mA range. Secondary mechanisms for avalanche triggering can be attributed to the after-pulse (due to undesired trapped carriers) and dark current (carriers generated by thermal agitation in the sensitive area) [9,10]. Coupling with an external electronic interface allows the SPAD to operate in the Geiger mode, which is an on–off-switching mode triggered by incident photons [11,12]. The operating phases of the photodetector are described as the triggering of the avalanche (on state) beyond the breakdown voltage (V_B_) and its reset to the previous biasing voltage V_A_ (off state). Two distinct photons can be properly resolved if the time between the two states is sufficient to restore the device, thus bringing the SPAD to the quiescent biasing point value (i.e., quenching and recharge). However, the probability that two photons are correctly detected is finite and related to the time of arrival interval between them on the target. When two or more photons are very close to each other, a SPAD detector may detect them as a single pulse of higher intensity, (i.e., peak pile-up). In other words, the detector cannot clearly distinguish between individual photons when they are so close together and, thus, it records them as a single, higher amplitude signal. Specifically, tail pile-up can occur when a photon reaches the SPAD after it recovers its status (pulse decay), but without sufficient time to restore the reverse bias point. To address these limitations, and to realize even faster sensors, passive (PQCs) and active quenching circuits (AQCs) were investigated. PQCs use simple configurations based on a single resistor to reduce the recovery time (typically in the µs range). When a lower dead time is needed, AQCs are used to detect the triggering of avalanche multiplication, limiting the total charge that runs through the SPAD down to a few ns [13,14]. AQCs allow control of the avalanche current and the dead time (the phase during which the SPAD is not sensitive), thus enabling researchers to accurately account for single photons, in the order of millions of counts per second. The pole-zero compensation technique is used to modify the frequency response of the circuit, reducing the impact of the detector capacitance and resistance, which cause phase variations and amplitude fluctuations in the detected signal. In SPAD circuits, the pole is due to an RC network produced by the connection capacitance and load resistance of the SPAD. The pole slows down the circuit’s response, resulting in a larger and less distinct pulse that is more difficult to detect accurately. To counteract this, a zero, designed with a time constant corresponding to that of the unwanted pole, is introduced. The result is a faster and more accurate signal response that closely follows the ideal current pulse generated by the SPAD at the time of photon detection. In this work, a monolithic avalanche photodetector was designed and simulated. To emulate the SPAD behavior and expedite the electronic interface design, a nanosecond pulse generator based on an avalanche transistor was designed and fabricated, allowing easy control of the signal shape by changing external discrete components in order to generate comparable signals. Moreover, we designed and fabricated an electronic interface based on pole-zero compensation to increase the resolution time between two consecutive photons in order to compensate for the limits of PQCs (i.e., the use of a high-value ballast resistor) [15,16]. The designed interface is expected to reduce dead time, thereby improving SPAD performance.

## 2. Materials and Methods

### 2.1. SPAD Design and Modeling

**Figure 1:**
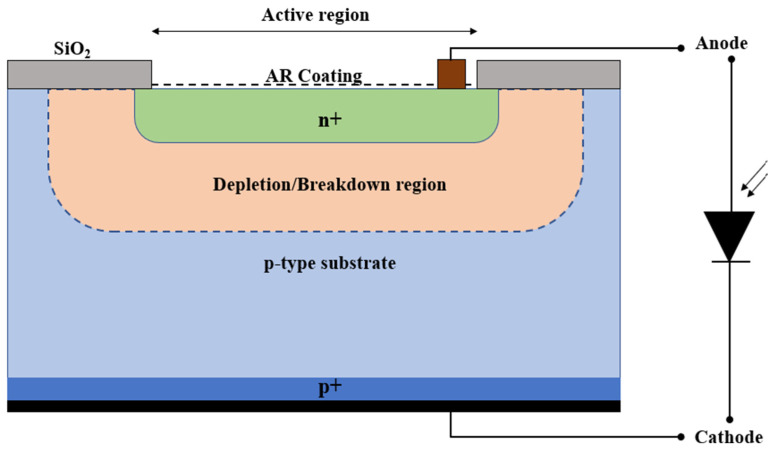
Cross-sectional view of the designed SPAD.

The structural configuration of the single-photon avalanche diode (SPAD), depicted in Figure 1, is characterized by a p-substrate/n+ region, achieved through the introduction of n-type dopants into a p-type silicon wafer [17].

Contact pads cover the front and the back surfaces of the device, while an anti-reflective coating is deposited on the active region [18]. Additionally, an insulating silicon oxide layer is deposited onto the inactive region to allow for the fine-tuning of speed and responsivity through adjustments in substrate thickness [19]. The SPAD demonstrates functionality in the forward, reverse, and reverse breakdown regions [20]. Avalanche events occur when the reverse bias voltage surpasses a critical threshold, leading to the generation of charge carriers through incident photons. To analyze the behavior of the proposed SPAD, Geiger mode simulations were employed, enabling the derivation of post-processing avalanche initiation probabilities based on electric field solutions. The breakdown voltage was calculated by integrating the ionization rates along the electric field lines from the anode to the cathode. The following equations describe avalanche initiation in the Geiger mode for the SPAD [21], where P_e_(x,y), P_h_(x,y), and P_p_(x,y) represent the probabilities of the electron, hole, and electron–hole pair initiation, respectively, at a coordinate (x,y):

dP_e_(x,y)/ds(x,y) = (1 − P_e_(x,y))α_e_ P_p_(x,y)

dP_h_(x,y)/ds(x,y) = (1 − P_h_(x,y))α_h_ P_p_(x,y) (1)

P_p_ = P_e_(x,y) + P_h_(x,y) − P_e_(x,y)·P_h_(x,y)

where α_e_ and α_h_ represent the electron and hole initiation rates, respectively, and s(x,y) indicates the distance along the field line. The joint probability signifies that the generation of an electron–hole pair at x at (x,y) will trigger an avalanche. When simulating the SPAD in Technology Computer-aided Design (TCAD), the Geiger mode simulations during the bias ramp were conducted by probing a point that was close to the center of the device.

**Figure 2:**
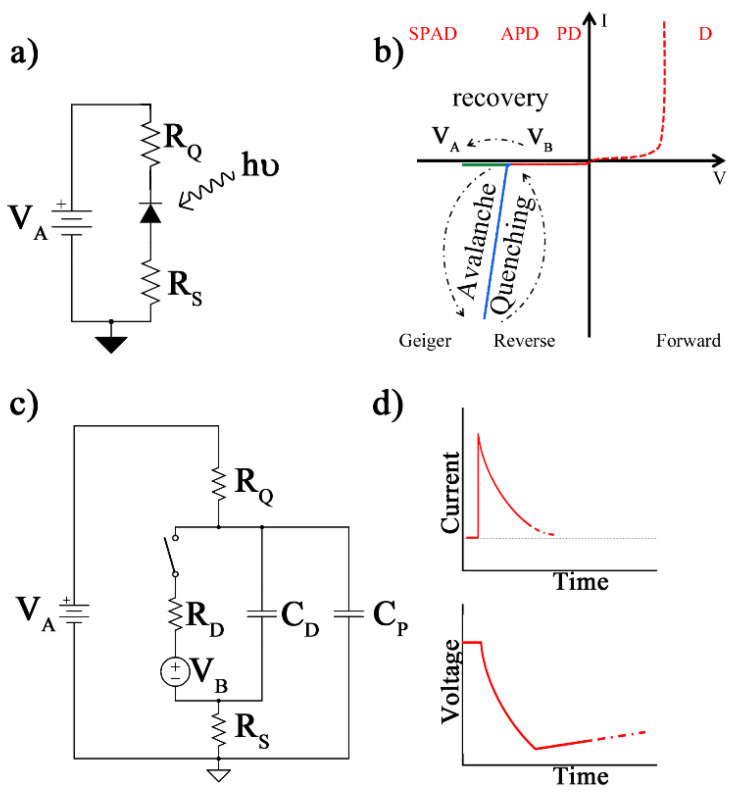
(**a**) Basic passive quenching circuit for a SPAD: (**b**) the relative working phases, (**c**) the voltage mode configuration and equivalent electrical model, (**d**) characteristic current flowing in the SPAD during avalanche triggering and voltage recovery to VA (not to scale).

A basic PQC, designed to bias the SPAD at a reverse voltage V_A_ through the so-called quenching resistor, R_Q_, is shown in Figure 2a [22,23].

A low-value resistor, R_S_, can be provided to obtain a voltage-related output pulse from the circuit. Concerning other junction-based devices (e.g., diode, D, photodiode, PD, avalanche photodiode, APD, and SPAD), the working phases of the SPAD are illustrated in Figure 2b. After avalanche multiplication is triggered by the incident photon, the quenching phase allows the device to lower the SPAD voltage to the breakdown before restoring its initial reverse voltage (V_A_) [24]. The basic electrical model, which describes its behavior, is shown in Figure 2c, where a switch is configured to model the incident photon that triggers the avalanche, the characteristic breakdown voltage, V_B_, and the internal resistance, R_D_. The capacitance of the junction and the parasitic capacitance to the ground are modeled as C_D_ and C_P_, respectively. As soon as the photon is detected on the SPAD, the switch closes, and the current increases rapidly, up to its maximum, while still maintaining the reverse voltage to V_A_ (no current flows through the quenching resistor, R_Q_) [25].

The increase in the diode increase lasts a few picoseconds, and the subsequent decrease defines the quenching phase (Figure 2d). The maximum current value in the SPAD is I_P_ = (V_A_ − V_B_)/R_D_, while its final value is I_F_ = (V_A_ − V_B_)/(R_D_ + R_Q_ + R_S_), and this transient is ruled by a time constant, τ_q_ ≈ RD∙(C_D_ + C_P_). The difference (V_A_ − V_B_) indicates excess polarization and influences avalanche triggering and the response time of the SPAD.

The voltage drop on R_Q_ increases and, therefore, the voltage on the SPAD reaches its final value V_F_ = V_B_ + (I_F_∙R_D_). Subsequent to the opening of the switch, the voltage across the diode returns to V_A_, with a recovery time τ_r_ = R_Q_∙(C_D_ + C_P_) (Figure 2d). At the end of this phase, the photodiode can detect a new photon with proper resolving time, thereby avoiding pile-up. This phase is usually much longer than the quenching period and establishes the response time of the SPAD [26].

### 2.2. SPAD Model and Electronic Interface

**Figure 3:**
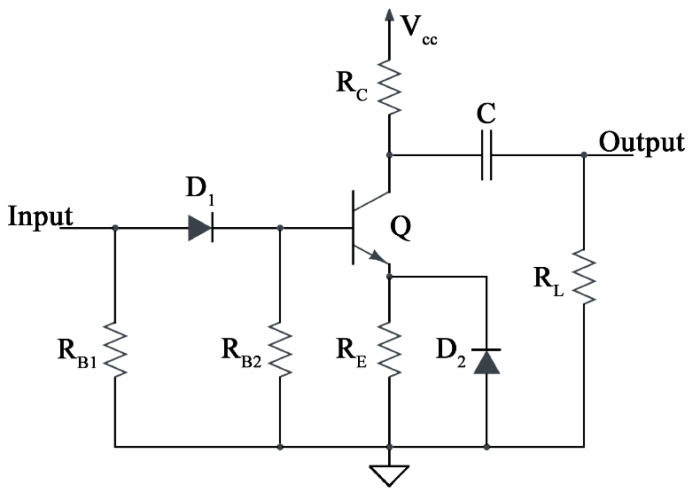
The avalanche transistor-based pulse generator.

A nanosecond pulse generator was designed to model the distinctive characteristics of the SPAD. It was based on an avalanche transistor for the generation of nano-/sub-nanosecond rising edge pulses, and its signal shape can be easily controlled by changing the components of the circuit, as shown in Figure 3. The capacitor, C, is charged to V_CC_ through the resistor, R_C_, and when the transistor is base-triggered, the stored charge flows through Q, R_E_, and D_2_. If the input is sufficiently high, a pulse is forced on the base through D_1_, which disconnects the input when the base voltage rises above the amplitude of the trigger. In this phase, the transistor is forced to sharply increase the collector current in the avalanche region with a rapid discharge of C, after which it reaches the cut-off region.

**Figure 4:**
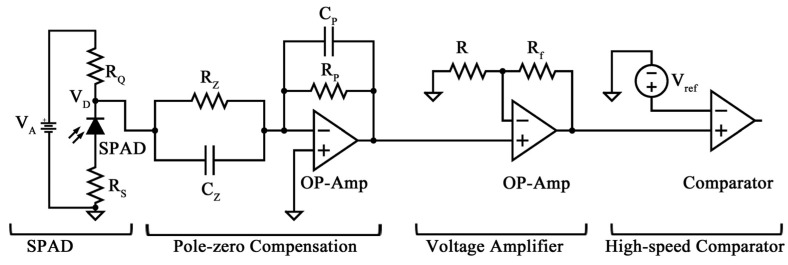
The electronic interface composed of the SPAD and passive quenching circuit (quenching resistor RQ), a pole-zero compensation stage, a voltage amplification stage, and a high-speed comparator.

The collector current peak depends on the avalanche multiplication factor, and the output signal can be measured in R_L_. The pole-zero compensation technique is a solution to this problem that is based on the elementary principles of frequency response. In fact, the pole of the SPAD can be canceled by a zero at the same frequency (ideally generating a unity impulse response). Then, the desired decay time can be set by introducing a pole at the desired frequency, reducing the overall decay time and lowering the saturation of the preamplifier circuits of the channel. Although the shape changes, no amplitude information is lost. The proposed solution, as shown in Figure 4, starts from the acquisition of SPAD voltage (V_D_), first implementing a pole-zero compensation on a slower pole (stable pole), then introducing a proper zero with the same time constant as that of the SPAD (i.e., τ_r_). The pole-zero compensation is achieved through the R_Z_ and C_Z_ network, by setting the value of R_Z_ = R_Q_ and C_Z_ = C_D_ + C_P_. Then a higher frequency f_P_ = 1/2πτ_P_ pole with τ_P_ = (R_P_·C_P_) is introduced, providing a shaping of V_D_ on the output (V_O_), which is characterized by a faster exponential decay. The transfer function of the shaper stage is as follows:

(2) $$\frac{V_{o1}}{V_{D}}=\frac{R_{P}}{R_{Z}}\frac{\left(1+{sR}_{Z}C_{Z}\right)}{\left(1+{sR}_{P}C_{P}\right)}\frac{\left(R_{f}+R_{P}\right)}{R}$$

where R_f_ and R are used to set the gain, while V_ref_ is used as the voltage reference for the comparator with respect to V_o1_, thereby obtaining output pulse (V_o2_) related to incoming photons.

The shaped signal is then amplified to adapt the amplitude levels before counting the incoming photon through a high-speed comparator. The voltage shape is consequently different, although the information can be retrieved without any loss.

This result occurs because the R-C compensation network introduces a zero with the same time constant as the photodiode and a pole related to a lower time constant; therefore, the C_D_ charge decays faster, and the use of an R-C compensation network with a photodiode changes the shape of the voltage to improve response time without losing the information content of the signal. The inclusion of a zero and a pole in the frequency response of the circuit leads to faster charging and discharging of the photodiode capacitance. Consequently, system performance is improved, and design, fabrication, and testing times are reduced, as reported in Section 3.

## 3. Results

The development of silicon-based SPADs can be used for the detection of photons in the visible spectral region up to ~1000 nm. For shorter wavelengths, alternative semiconductor materials can be used (e.g., InGaAs, Ge(Sn), and Mercury Cadmium Telluride, MCT). However, the proposed electronic interface can be used also in these cases. It is possible to acquire precise parameters for the model with the help of Geiger mode simulation in TCAD. The designed two-dimensional (2D) SPAD structure was simulated in TCAD employing commercial 0.18 μm CMOS process parameters.

Two-dimensional device simulation was performed in cylindrical coordinates (r,y) incorporating Shockley–Read–Hall recombination, low-field mobility, impact ionization, energy balance transport, Geiger models, etc.

A spontaneous avalanche occurs at the highest electric field near the edge of the active area, and a high impact generation rate is found to be concentrated in the avalanche region. The avalanche breakdown occurs naturally at the point of the strongest field, usually located at the edge of the active region. However, in the Geiger mode, the voltage is determined where a carrier generated by a photon triggers an avalanche process anywhere within the SPAD, not only at the edge of the anode. In this study, we investigated a specific point near the center of the device while gradually increasing the bias. We conducted simulations in the Geiger mode to demonstrate the probabilities of electrons, holes, and their joint probability in the proposed SPAD.

**Figure 5:**
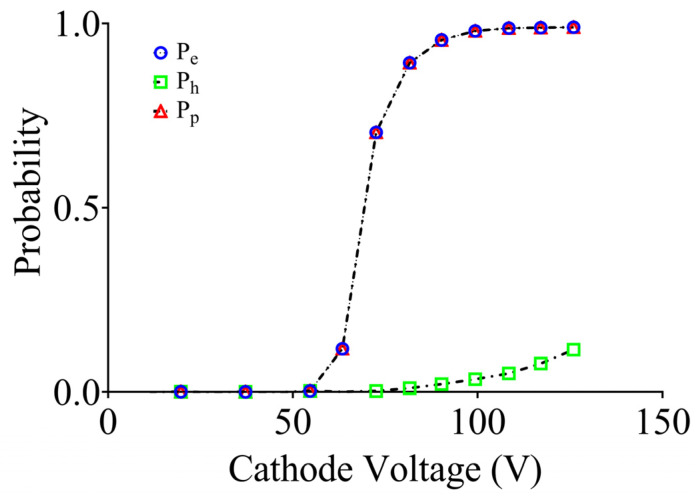
Electron, hole, and joint probabilities versus reverse voltage.

**Figure 6:**
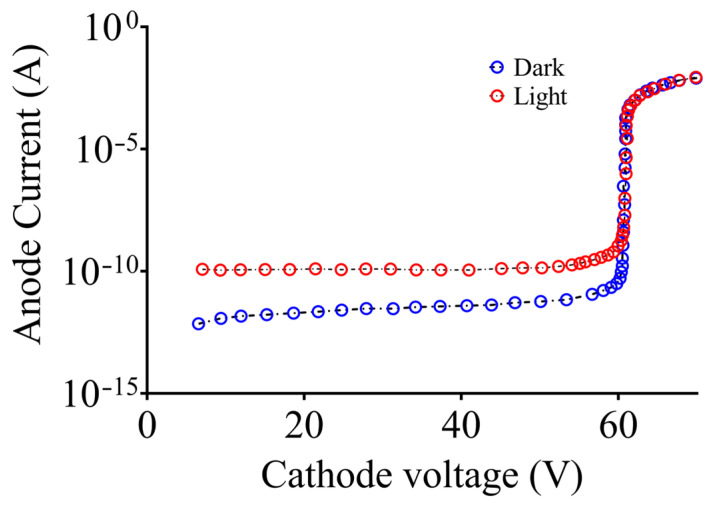
Current versus voltage (I–V) characteristics of SPAD under dark and light conditions.

The electron (P_e_), hole (P_h_), and electron–hole pair (P_p_) probabilities are presented in Figure 5, which illustrates the probabilities of the carrier entering into the multiplication region and triggering an avalanche. Electrons are responsible for initiating a higher number of ionizing events than holes. Since αe > αh in silicon, the likelihood of breakdown for pure electron injection at any reverse bias above the breakdown voltage is higher than that of pure hole injection. The current versus voltage (I–V) characteristics of the SPAD are approximated by ramping the cathode voltage up to 80 V; when the diode subsequently breaks down at 63 V, the Geiger mode kicks in, and the simulated breakdown voltage exhibits good agreement with the experimental findings. The SPAD displays various current characteristics below and above the breakdown voltage while operating in reverse bias, as depicted in Figure 6. Under dark conditions, the SPAD displays an extremely low dark current, of a few picoamperes, when a reverse bias below the breakdown voltage is applied.

The current, however, increases significantly, due to impact ionization and multiplication, when a voltage above the breakdown voltage is applied to the device.

The current quickly reaches its maximum as a result of the space–charge effect and the series resistance. Under the illuminated condition, a similar phenomenon is observed. Low photogenerated currents, of a few nanoamperes, flow below the breakdown voltage.

The current rapidly increases, as the reverse bias exceeds the voltage, and quickly reaches saturation for the same reason as in the dark condition. Resistance is added serially in the barrier region and the space–charge layer to determine the diode resistance (R_D_). A smaller sensing area and a thicker depletion region increase R_D_, which typically ranges from 100 Ω to a few kΩ. Since the SPAD employed in this study has a large sensing surface and a thick barrier region, the diode resistance is assumed to be 1 kΩ. A large R_Q_ in SPADs allows for adequate quenching and a faster discharge, with less jitter. Thus, R_Q_ is normally maintained around 100 kΩ. The initial simulation of the SPAD (in Figure 2c) was performed, from triggering to self-quenching of the avalanche, using an analogy between the photodiode itself and an avalanche transistor-based circuit (Figure 3).

**Figure 7:**
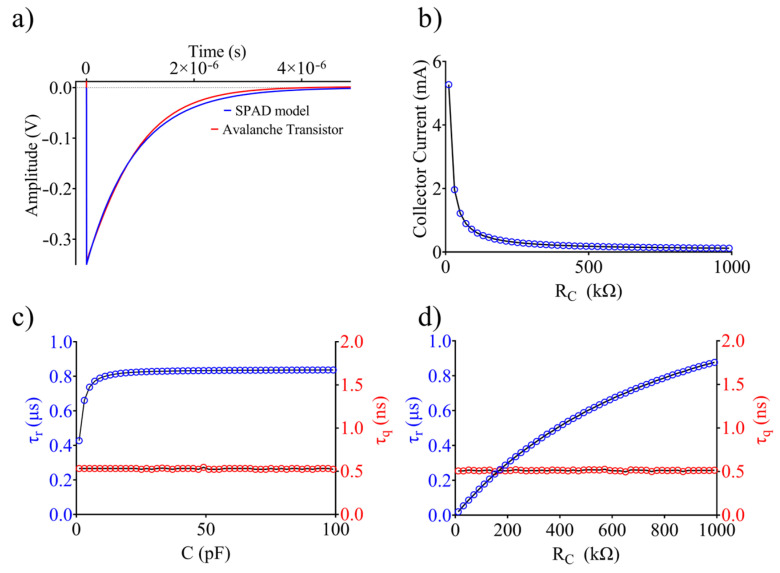
Time response to a single trigger pulse of the SPAD and pulse generator (**a**). Current level flowing into the collector when varying resistor R_C_ (**b**). Evaluation of recovery time constant and quenching time constant when varying C (**c**) and R_C_ (**d**).

In Table 1, the simulation data from the SPAD model and the pulse generator are reported. The switching part of Figure 2c was implemented using an NMOS transistor. In the quiescence state, the switch is open and the output is fixed to V_A_ = 65 V. When a positive pulse is driven simulating an incoming photon, avalanche multiplication is triggered. The current flowing into the photodiode reaches a maximum value of I_P_ = 65 mA and approaches its final value I_F_ = 640 µA according to the characteristic time constants τ_q_ = 10 ns and τ_r_ = 1 µs, respectively (Figure 7a). A current level higher than 100 µA ensures a self-sustained avalanche process [27].

In order to ensure flexibility and prompt design implementation, the SPAD behavior (e.g., V_D_) was simulated using a pulse generator based on an avalanche transistor, as shown in Figure 3, which exploits the transition from high to low impedance conditions in the V_CE_-I_C_ region. The transistor is initially off (negligible current flowing into Q through R_C_, R_E_, and D_2_), and the voltage across the capacitor C is set to 110 V (beyond the breakdown voltage, BV_CE_). Very few models are available for transistors operating in the avalanche region; thus, numerical simulations are the most-used analysis technique [28,29]. Nevertheless, Roehr proposed a differential model for a transistor working with BV_CE_ < V_CE_ < BV_CB_, which includes a resistive element, r_A_, shunted by capacitance CA [30].

Both elements, evaluated between the collector and the emitter, are nonlinear and negative. Working at high I_C_, r_A_ approaches zero and C_A_ approaches minus infinity. Conversely, at low I_C_, r_A_ approaches infinity, while C_A_ approaches the collector–base capacitance since the emitter is open [31]. Thus, when a positive input is given into the base, the transistor is forced to switch from the off to the on state, a condition which is characterized by a region of negative resistance. The capacitance, C, discharges onto the equivalent resistance of (r_A_ + R_E_ + R_L_)//R_C_, thereby generating a proportional voltage on R_L_.

The advantage of using such an avalanche transistor is the possibility of obtaining a high-frequency, large current pulse source. The rise time of the pulse is limited by the turn-on time of the transistor. However, attention must be paid to choosing such components. A collector resistance in the range of 1 kΩ–1 MΩ results in a maximum collector current level of 5 mA down to 120 µA, which is sufficient to sustain the avalanche process (Figure 7b). Moreover, the n-p-n transistor model must take into consideration the collector–base (C_JC_) and base–emitter (C_JE_) transition capacitances. Their values are provided at zero bias and are strongly influenced by the bias of the transistor, resulting in an overall base–emitter and base–collector capacitance interacting with C.

The simulated electrical model of a bipolar transistor includes two base–emitter capacitances and two base–collector capacitances, which are considered for the transit time (C_BEτ_, C_BCτ_) and junction (C_BEJ_, C_BEJ_) capacitances. In the actual arrangement, during the off state, the base–emitter junction results as weakly reverse-biased, while the base–collector junction is strongly reverse-biased. Therefore, the overall capacitive effect is due to the contribution of the depletion base–collector capacitance, which is reduced as the reverse voltage increases [32].

As shown in Figure 7c, the choice of C influences the recovery time constant τ_r_, since, when Q is off, the overall capacitance between the collector and the ground should also be considered. A capacitance higher than 10 pF results in a substantially constant, τ_r_ = 830 ± 8 ns. In this range, as in the case of a SPAD, the junction capacitances rule the dynamics of the circuit. Contrary to what happens in the photodiode, for C > 10 pF, the time constant can be more easily controlled externally. As expected, in all the cases, C does not influence the quenching time constant (τ_q_ = 0.53 ± 0.01 ns), as shown in Figure 7c. In addition, the collector resistance influences the recovery time constant, since it is involved in the discharge of the charge stored in the off phase of the transistor, as shown in Figure 7d. Finally, the quenching time is not affected by R_C_ variations (τ_q_ = 0.51 ± 0.01 ns) in this case.

Thus, the working principle of the SPAD can be substantially similar to that of an avalanche-based transistor working as a pulse generator. As shown in Figure 7a, a high accordance can be observed in the simulations of both circuit models.

The transistor-based generator also includes an adjunctive bypass capacitance and a voltage divider to adapt the voltage level for the next stage. Even though the time responses of the SPAD and the pulse generator are quite similar, the electrical models used for the simulation of both circuits are completely different. Consequently, the zero introduced for compensation should be specifically adapted to match the two models.

In Table 2, the data used for the simulations of the compensated SPAD and the pulse generator circuits are reported.

The op-amp LMH6702 was chosen due to its very wide frequency band (1.7 GHz) with high unity gain stability at exceptional speed (rise/fall time of 1.7 ns) and provision for additional external compensation. The ADCMP561 comparator is characterized by a 700 ps propagation delay and an overdrive dispersion < 75 ps.

**Figure 8:**
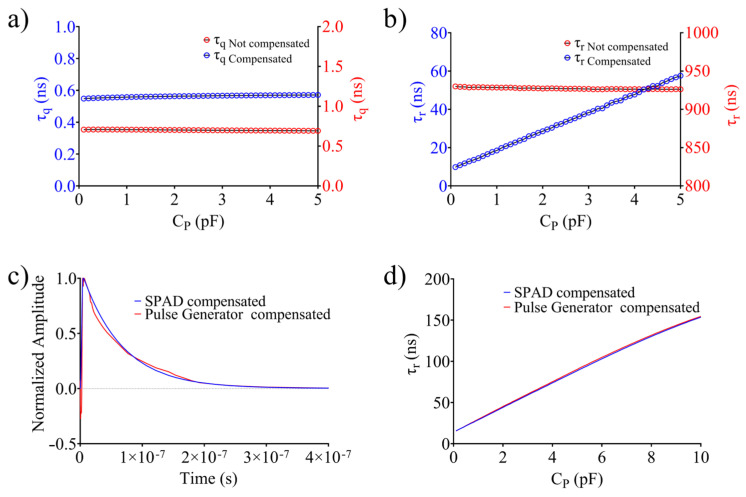
Quenching time constant before and after compensation with varying capacitor CP (**a**). Recovery time constant with varying capacitor CP before and after compensation (**b**). Comparison of the output voltage (**c**) and recovery time constant (**d**) between the SPAD model and the avalanche-based pulse generator.

Figure 8a shows the quenching time constant, evaluated before and after the incorporation of the electronic interface for pole-zero compensation by varying C_P_ in the range of 0.1–5 pF, although C_P_ does not have a significant influence on τ_q_. However, a constant increase in the quenching time constant from 0.53 ± 0.01 ns up to = 0.70 ± 0.01 ns was observed, which may be due to the two op-amp-based stages. As evident in Figure 8b, the recovery time is strongly influenced by the pole-zero compensation circuit. By varying CP in the same range (i.e., 0.1–5 pF), the recovery time constant can be lowered from 927.3 ± 1.1 ns (not compensated) down to 57.6 ns and 9.8 ns by lowering C_P_ to 0.1 pF. The comparison between the simulated output voltage of the SPAD and the avalanche-based pulse generator (using data from Table 2), is illustrated in Figure 8c, demonstrating substantial agreement between the two responses. The same findings can be observed when analyzing the recovery time of the two models by varying C_P_ (Figure 8d). The above-discussed simulation results have been experimentally verified according to the data reported in Table 1 and Table 2.

**Figure 9:**
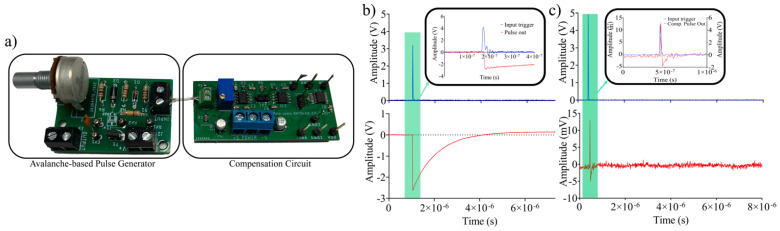
Discrete printed circuit boards of the avalanche-based pulse generator (**left**) and the pole-zero compensation circuit (**right**) (**a**). Experimental input trigger to the avalanche-based pulse generator, and the non-compensated output voltage (**b**). Experimental input trigger to the avalanche-based pulse generator, and the compensated output voltage Vo (**c**).

The discrete circuit board of the avalanche pulse generator and the pole-zero compensation circuit are shown in Figure 9.

The circuit was realized using the discrete components reported in Table 2, on a double-layer printed circuit board (Figure 9a). A positive trigger signal of 5 V was delivered, using a function generator (Tektronix, Beaverton, OR, USA, AFG3102), with a duration of 8 ns and rise and fall times of 5 ns each. The output signal was acquired using a digital oscilloscope Tektronix DPO3054. Figure 9b shows the input trigger and the output signal, Vo, without compensation.

It is evident from the figure that the input signal is mostly imposed by the dynamics of the function generator, with a pulse width of about 18 ns. At the same time, the non-compensated output voltage evidenced a τ_r_ of approximately 1 µs, in accordance with the previous simulation shown in Figure 8c. In addition, a lag time of about 6 ns exists between the input pulse and the output. Figure 9c shows the effect of the pole-zero compensation circuit, in which the output pulse signal is 180 degrees out of phase, according to the circuit topology, and is characterized by a fall time of 15 ns, a rise time of 7 ns, and a maximum voltage swing of 20 mV for the pulse. The amplitude level can be adapted by adjusting the gain of the non-inverting voltage amplifier stage, as illustrated in Figure 4 (set to 0 dB), which further results in increased recovery time for incoming photons by approximately 100 times. Recent advances in 3D-stacking [33,34,35,36] show the way to the next generation of high-performance SPAD imagers, resolving the trade-off between SPAD and optimization of processing electronics, as the upper and the lower layers can be implemented in different technologies, one optimized for SPADs and the other for high-functionality, low-power electronics. In SPAD, the problem of pulse accumulation must be analyzed in the case of high-input speed applications. This problem can be addressed by implementing pole-zero cancellation circuits. An example of this type of circuit can be found in, where the pole-zero compensation was implemented with twenty PMOS transistors and a 24-pF capacitor. Furthermore, to address the pile-up problem, pole-zero compensation eliminates undershoot and allows higher speed counting [37]. In recent years, emerging technologies based on 3D stacking are paving the way for a new generation of SPAD detectors with short dead times, low pixel pitch, high pixel count, and the ability to integrate on-chip image processing, which historically has been the parameters that have most limited the applicability of SPAD arrays [38,39,40]. The proposed compensation circuit has shown that the larger time constant of SPAD can be reduced and that the signal distortion due to the nonideal zeroing of the poles is minimal and mainly concerns an under-elongation of the compensated signal (Figure 9c). This circuit also can be easily integrated into a monolithic device, which is of considerable interest in systems with multiple detectors.

## 4. Conclusions

Geiger-mode semiconductor devices enable the detection of single photons with high temporal resolution. For this reason, they are valuable in applications such as quantum key distribution, fluorescence lifetime imaging, and time-resolved spectroscopy that require extreme sensitivity and accurate timing. Some studies have used SPAD devices for the time-of-flight technique. In fact, the devices underwent a rigorous design process to improve the signal-to-noise ratio and achieve exceptional sensitivity, even for single-photon counting. The use of SPADs for the detection of single photons has achieved promising performance in terms of efficiency and effectiveness. SPADs allow for the extraction of a signal that marks the arrival time of a single photon, which is a principal feature in an application based on timing. The electrical characteristics of such devices strongly depend on the design constraints and fabrication processes; furthermore, the design of a SPAD is a critical process that is time-consuming, resulting in high variability in its characteristics. In this work, the development and optimization of an electronic interface based on pole-zero compensation were investigated, to improve the count rate of SPADs. Moreover, a nanosecond pulse generator based on an avalanche transistor was investigated as a pseudo-SPAD, used to generate comparable voltage pulses in order to expedite the electronic interface design, thus avoiding excessive latency between the design and the fabrication processes. The results confirm that the SPAD and avalanche transistor-based electronic interface are in strong accordance, providing similar output pulses as in the case of an illuminated SPAD and as a result can help reduce the design, fabrication, and testing times of SPADs. Moreover, an AQC based on the pole-zero compensation technique demonstrated significant improvement in the dynamics of the biased SPAD, reducing the recovery time by approximately 100 times.

## Figures and Tables

**Table 1:** Experimental data for SPAD and pulse generator simulation.

SPAD Model	Pulse Generator
V_A_ = 65 V, R_Q_ = 100 kΩ	V_A_ = 110 V, R_Q_ = 800 kΩ
R_D_ = 1 kΩ	R_B1_ = R_B2_ = 130 Ω, R_E_ = 100 Ω
V_B_ = 60 V, C_D_ = 9 pF	C = 1 μF, R_L_ = 500 kΩ, Q = ZTX415
C_P_ = 1 pF, R_S_ = 100 Ω	D_1_ = D_2_ = 1N4148

**Table 2:** Simulation data for pole-zero compensation circuit.

SPAD Model Compensator	Pulse Generator Compensator
C_Z_ = 10 pF, R_Z_ = 100 kΩ	C_Z_ = 1.5 pF, R_Z_ = 350 kΩ
C_P_ = 1 pF	C_P_ = 1 pF
R_P_ = 35 kΩ, R = 237 Ω	R_P_ = 35 kΩ, R = 237 Ω
R_f_ = 237 Ω	R_f_ = 237 Ω
Op-amp: LMH6702	Op-amp: LMH6702
Comparator: ADCMP561BRQ	Comparator: ADCMP561BRQ

## Data Availability

Data are contained within the article.
